# Applicability of Machine Learning in Behavioural Monitoring of the Red Panda (*Ailurus fulgens*) in Zoos

**DOI:** 10.3390/ani16081165

**Published:** 2026-04-10

**Authors:** Amalie M. Worup, Anne S. Sonne, Jeppe Kudahl, Johanne H. Jacobsen, Sussie Pagh, Thea L. Faddersbøll, Cino Pertoldi

**Affiliations:** 1 Department of Chemistry and Bioscience, Aalborg University, Frederik Bajers Vej 7H, 9220 Aalborg, Denmark; aworup22@student.aau.dk (A.M.W.); asonne22@student.aau.dk (A.S.S.); jkudah21@student.aau.dk (J.K.); jjac21@student.aau.dk (J.H.J.); sup@bio.aau.dk (S.P.); 2 Aalborg Zoo, Mølleparkvej 63, 9000 Aalborg, Denmark; tlf@aalborgzoo.dk

**Keywords:** object detection, behaviour categorization, LabGym

## Abstract

Welfare assessment for the endangered red panda (*Ailurus fulgens*) in captivity requires systematic behaviour monitoring, yet traditional direct observation is often limited by observer subjectivity and time constraints. This study evaluates the feasibility of employing machine learning (ML) to automate behavioural monitoring of a red panda in a complex, mixed-species enclosure at Aalborg Zoo, Denmark. Using video data from cameras in the enclosure of the red panda, and the ML model LabGym for animal detection and behavioural categorisation, models were trained to analyse activity patterns of the red panda. The results demonstrate that, while the behaviour categorizer is a promising tool with high classification confidence, the overall system effectiveness is currently limited by the object detector’s performance in a naturalistic environment. Challenges such as environmental obstructions (e.g., rocks, foliage, and trees) and the animal’s camouflage contributed to a significant amount of unclassified time, which may affect the overall assessment of behavioural distribution. We conclude that, while ML holds potential for non-invasive behaviour monitoring, its application in complex zoo settings requires improved detection capabilities to be fully reliable. Future iterations of this system could be enhanced by complementing standard object detection with pose estimation frameworks. Implementing alternative labelling strategies or background subtraction methods could additionally mitigate the detection challenges posed by environmental obstruction.

## Simple Summary

Ensuring high welfare standards for captive animals in zoos requires consistent behavioural monitoring, yet traditional manual observations are time-consuming and often miss nocturnal activity. This study explored the potential of artificial intelligence, specifically machine learning, to automate the tracking of red panda behaviour within a complex zoo habitat. By training a computer model to recognize activities such as resting, feeding, and grooming, we aimed to create a non-invasive tool for 24 h welfare assessment. Our findings reveal that, while the technology is highly accurate at identifying specific behaviours once the animal is located, its overall effectiveness is currently hindered by the red panda’s natural camouflage and the dense foliage of modern enclosures. This resulted in gaps in the automated records compared to human observers. We conclude that, while machine learning offers a promising future for animal care, further refinements are needed to navigate the physical complexities of naturalistic zoo environments. Ultimately, this technology could provide zookeepers with a vital “early warning system” to detect subtle changes in animal health, ensuring higher welfare standards for endangered species in captivity.

## 1. Introduction

The red panda (*Ailurus fulgens*) is listed as “Endangered” on the IUCN Red List. Red pandas are arboreal mammals inhabiting bamboo understories and forests in Nepal, India, Bhutan, Myanmar, and China. Due to habitat loss and fragmentation, many of the existing red panda populations are threatened by low densities within fragmented forest patches. It is estimated that the red panda population has declined by 50% within the last three generations, a trend that appears to be continuing [1,2,3].

As a result, international ex situ conservation organizations, such as the World Association of Zoos and Aquariums (WAZA) and the European Association of Zoos and Aquaria (EAZA), which manages the EAZA Ex-situ Programmes (EEPs), play a crucial role in maintaining genetic diversity and supporting the long-term conservation of the species [4,5]. Newly added regulations from the WAZA 2023 Animal Welfare Goal dictate that all WAZA member zoos are required to have and go through an animal welfare evaluation process which must include certain elements decided by WAZA. As part of such a process members are required to provide evidence that monitoring of animal welfare is happening systematically and continually. Monitoring of animal behaviour can be used as part of a welfare assessment through frameworks such as the ’Five Domains’ model consisting of nutrition, environment, physical health, behaviour and the mental domain [6,7,8]. Assessing these domains in a holistic context allows for a comprehensive evaluation of animal welfare [8].

### 1.1. Current Assessment of Animal Welfare

The welfare of captive animals is essential for the success of conservation breeding programs, and for fulfilling ethical obligations. New regulations and obligations from organisations such as EAZA and WAZA have strengthened zoos’ obligations to ensure the proper welfare of animals [4,5].

Zoos traditionally determine welfare through a combination of methods such as direct behavioural observations, measurement of hormone levels, and recording of health and environmental parameters [9,10,11]. However, reliance on direct behavioural observations presents significant limitations. Direct behavioural observations are both time-consuming and often limited to daytime observations [11]. Furthermore, direct observations of animal behaviour exhibit a challenge in the subjectivity of the observer, as even with the inclusion of inter-observer reliability testing there have been found to be significant gaps between the behaviour categorisation of multiple observers [12,13].

Reliance on direct behavioural observations is particularly problematic for species with crepuscular or nocturnal circadian rhythms, as their peak activity periods are often missed due to the absence of zoo keepers, researchers or other observers [11,14]. For example, the red panda, both in the captive and wild environment, is most active at dawn/dusk and around midnight [3,15]. This limitation highlights the need for a continuous and non-invasive method for monitoring behaviour in captive animals [16,17].

### 1.2. The Integration of ML in Biological Studies

Recent advancements in ML have revealed several possible applications in biological studies, by offering efficient and automated solutions to the limitations of traditional observational methods [17,18]. This technological approach enables mass-data utilization and real-time tracking of animal behaviours, which directly aids in the fulfilment of animal welfare goals such as the WAZA 2023 Animal Welfare Goal [7,19]. While ML is extensively applied in general object recognition and tracking, its application is still in the early stages in complex and species-specific behaviour analysis [20,21].

Studies on other species have demonstrated utility of ML, yet the specific challenges posed by a zoo environment remain significant. Technical challenges include the complexity of the enclosure structure, the difficulty in distinguishing between behaviours that look similar, the potential for false positives, and the risk of overfitting due to limited diversity in training data [19,22].

To address these challenges, this study aims to evaluate the efficacy of ML techniques for automated behavioural monitoring of red pandas in captivity and determine whether such a model can supplement the zookeepers’ and researchers’ regular behaviour observations. Ultimately, this study seeks to validate ML as a non-invasive tool for continuous welfare monitoring of red panda in zoos.

## 2. Materials and Methods

### 2.1. Subject and Study Site

**Figure 1:**
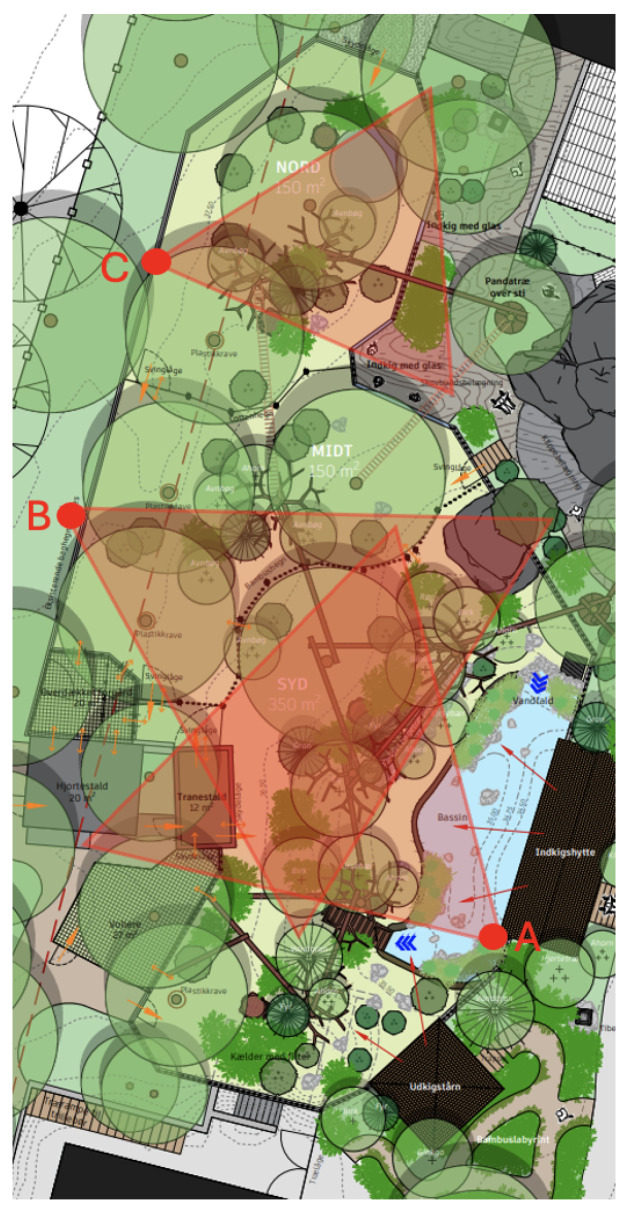
Layout of the red panda enclosure in Aalborg Zoo (*Illustrated by Kåre Thomas Jensen, on behalf of Aalborg Zoo*). Placement of the video capture security cameras (A, B, and C) is indicated by red dots, with corresponding vision cones showing an approximation of enclosure coverage. Field view of camera A, B, and C can be seen in Appendix A.

This study examined the behaviour of one captive male red panda housed at Aalborg Zoo, Denmark. This red panda was born in Prague in June 2021 and arrived at Aalborg Zoo in April 2022. The red panda enclosure is a relatively newly renovated habitat designed to promote complex dimensionality. The facility was officially inaugurated in June 2025. It was constructed to house a breeding pair of red panda and incorporates complex dimensionality with several options for climbing, including interspersed natural trees connected by logs, rope bridges, and elevated platforms throughout the outdoor area, as well as sleeping dens and a smaller indoor area (Figure 1). The enclosure also features a basin and is structured for cohabitation with a breeding pair of red-crowned cranes (*Grus japonensis*).

The red panda’s diet consists of plant-based feeding pellets for leaf-eating animals (DK Zoological), made from a blend of vegetables, grains and protein sources, in addition to fresh bamboo leaves, located at set points in the enclosure within view of potential audiences.

### 2.2. Data Collection

Observational data were collected between 20 September 2025 and 26 October 2025. Video was captured with three security cameras (Milesight MS-C2941-X30RPE/W, Xiamen, China, 1920 × 1080p, 15 fps) positioned at location A, B, and C within the enclosure (Figure 1).

Before model training and evaluation, an ethogram with relevant behaviours was created based on behavioural studies of the red pandas in Aalborg Zoo and literature detailing behavioural studies of captive red pandas [15,23,24]. The selected behaviours and definitions can be seen in Table 1. The behaviours were used to categorize behavioural examples for model training in LabGym [25]. Subsequently, manual observations were conducted as a basis for comparison to evaluate the accuracy of the trained model.

#### Inter-Rater Reliability

To ensure the consistency and reproducibility of the behavioural observations, an inter-rater reliability analysis was conducted. Before the final data collection, an iterative training process was employed to refine the ethogram.

Four independent raters scored a subset of the video data containing representative behaviours. Following the initial scoring, discrepancies were reviewed, and behavioural definitions were discussed and adjusted to minimize ambiguity. This process of scoring, discussion, and ethogram refinement was repeated until consensus was reached and the reliability coefficients met the criterion for minimum degree of agreement (defined as ICC > 0.75) [26].

The Intraclass Correlation Coefficient (ICC) was employed to assess inter-rater reliability, quantifying the degree to which different observers provided consistent and absolute measurements of the same behaviours. A high ICC value indicates that the observed behavioural distributions are reproducible and that the variance in the data is driven by actual animal activity rather than observer bias or measurement error.

The ICC was calculated based on the mean square estimates derived from an analysis of variance (ANOVA). The obtained ICC was computed by a single-rating, absolute-agreement, 2-way random-effects model using the following equation [27]:

(1) $$\mathrm{ICC}\left(2,1\right)=\frac{MS_{B}-MS_{E}}{MS_{B}+\left(k-1\right)MS_{E}+k\left(MS_{J}-MS_{E}\right)/n^{\prime }}$$

where $MS_{B}$ is the mean square between subjects (targets), $MS_{J}$ is the mean square for raters (judges), $MS_{E}$ is the mean square error, *k* is the number of raters, and *n* is the number of targets.

### 2.3. Model Training

**Figure A4:**
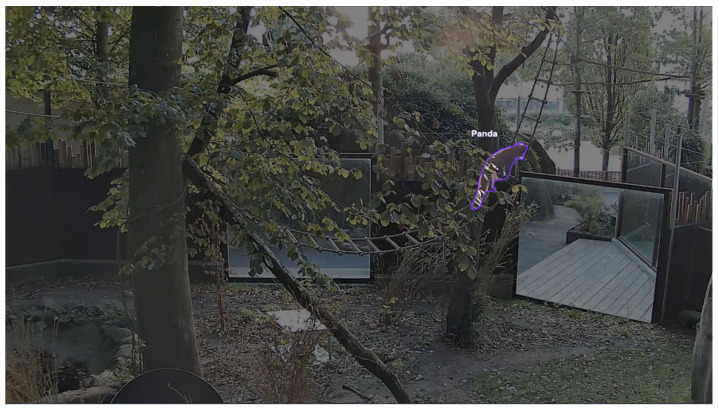
Example of polygon annotation for subject detection. Representative frame from the annotation workflow in Roboflow. The subject is delineated by a manually drawn polygonal mask (purple outline, labelled “Panda”).

To test the applicability of ML in the behavioural observation of the red panda, a combined software approach was utilized. LabGym (version 2.9.6) was used for frame sampling and object detector training. Roboflow [28] was used to annotate the images (Figure A4) and create the augmented dataset for object detection. Frames were systematically sampled at 5-s intervals from the collected video material. Due to the subject being frequently absent from the footage, the final dataset was curated to ensure the subject was present in at least 95% of all sampled images. The annotated images were augmented using horizontal and vertical flips and rotations (clockwise, counter-clockwise, and upside down) to increase the dataset’s size and variability. The complete dataset was partitioned into 90% training examples and 10% validation examples. An object detector was subsequently trained in LabGym with 10,000 iterations.

**Figure 2:**
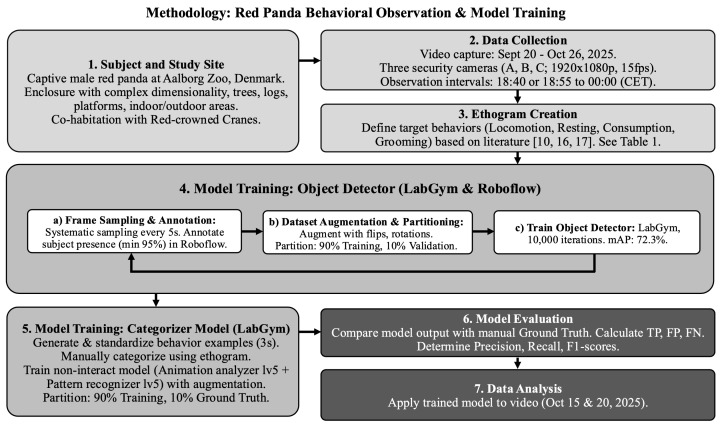
Schematic overview of the experimental methodology. The framework encompasses site selection and data acquisition, ethogram creation, and the iterative training and validation of ML models (LabGym and Roboflow Version 1.0) for automated behavioural analysis and evaluation [10,16,17].

**Figure A5:**
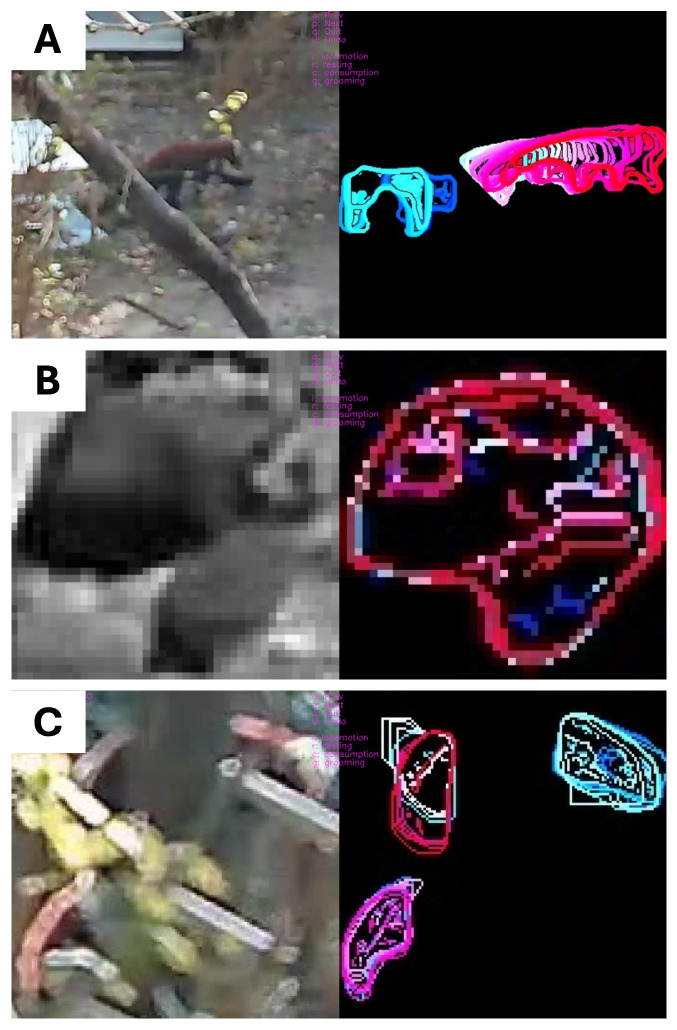
Representative behavioral examples used for model training. Paired images showing the video segment (left) and the corresponding LabGym segmentation masks (right), where the colour gradient represent temporal progression of the movement (blue indicating early and red indicating late frames). These manually validated clips served as the ground truth for training the categorizer model: (**A**) Locomotion, (**B**) Resting, (**C**) Grooming.

The detector was then used to detect and generate behaviour examples of the red panda (see Figure A5) to be used in training the categorizer model. The generated examples were standardized to a duration of 3 s to adequately capture continuous behaviours. Examples were then manually categorized into behavioural categories in accordance with the ethogram (Table 1). 90% of sorted behaviours were used for model training and 10% were kept as a ground truth for later model evaluation. The categorizer model was trained within LabGym using a non-interact model (Animation analyser lv5 + Pattern recognizer lv5), with default settings for input shape and augmentation (rotation, flipping, brightening, and dimming). Training was measured in epochs, representing the number of times the entire training dataset passed through the model. During this process, a loss function was utilized to quantify the difference between the model’s predictions and the ground truth labels, with the goal of minimizing this value to improve accuracy. The complete workflow, from data acquisition to model evaluation, is illustrated below (Figure 2).

### 2.4. Model Evaluation and Statistical Analysis

Evaluation of the model was performed by testing it against the manually annotated ground truth data. The performance of the object detector was evaluated using mean Average Precision (mAP), which calculates the average precision across all categories and spatial overlap thresholds. For the behavioural classification, True Positives (TPs), False Positives (FPs), and False Negatives (FNs) were calculated. From these results, the precision, recall, and F_1_-scores were determined for all behaviour types. These measures served as the primary indicators of the model’s efficacy in correctly sorting new behavioural observations. Precision represents the proportion of predicted positive observations that were actually correct, while recall measures the model’s ability to identify all relevant instances within the dataset. The F_1_-score provides a mean of these two metrics, offering a single balanced measure of the model’s overall performance.

Following the performance evaluation, the trained model was deployed to analyse the spatial distribution of behaviour and investigate how the different camera angles captured the subject’s activity. The analysis was conducted on video material from two distinct observation days (15 October 2025, and 20 October 2025) across the three camera zones (Figure 1). The observation period for this analysis ranged from approximately 18:40 to 00:00 (CET). This specific time interval was selected to align with the red panda’s expected peak activity levels in accordance with its circadian rhythm [3,15], thereby ensuring sufficient behavioural data for the comparison of camera zone efficacy.

All data processing and graphical renderings of results were conducted using RStudio (Version 2025.09.2+418).

## 3. Results

This study assesses the technical performance of the ML models, followed by a validation against manual ground truth data on red panda behaviour.

### 3.1. Reliability Analysis

The finalized ethogram demonstrated excellent inter-rater reliability across all measured behavioural categories. As shown in Table 2, the ICC values ranged from approximately 0.85 to 0.97, confirming that the behavioural definitions were sufficiently robust for data collection.

### 3.2. Model Training Results

The final detector model reached a mAP of 72.3%. Training was concluded at this stage as further increases in dataset size and training iterations yielded diminishing returns in mAP, suggesting that the model had effectively captured the available features within the constraints of the current dataset and environmental conditions.

**Figure 3:**
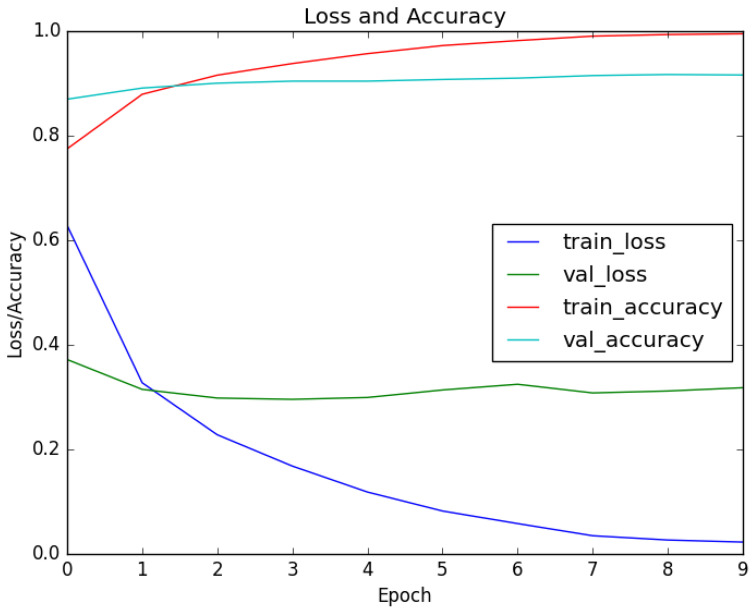
Progression of categorizer training including loss of validation and accuracy across epochs. The dark blue and green curves respectively show training and validation losses, where lower values indicate better model performance. The red and light blue curves respectively show training and validation accuracy, where higher values indicate better model performance.

The LabGym categorizer model completed training after 9 epochs, as accuracy plateaued near 1 and loss near 0. The evolution of these metrics is illustrated (Figure 3).

Precision, recall, and F_1_-scores were computed on the ground truth data to determine model performance across all behaviour types (Table 3). The categorizer showed good performance in categorizing all behaviour types with an overall accuracy of 76%. Consumption exhibited the lowest F_1_-scores, primarily driven by poor recall (56%) despite having the largest support (59 samples). Conversely, the locomotion category demonstrated the highest recall at 94%, indicating the model is highly effective at identifying true instances of movement. Performance for resting was the most balanced, achieving a robust F_1_-score of 84% through equally strong precision and recall.

### 3.3. Spatial Variance in Classification Confidence

**Figure 4:**
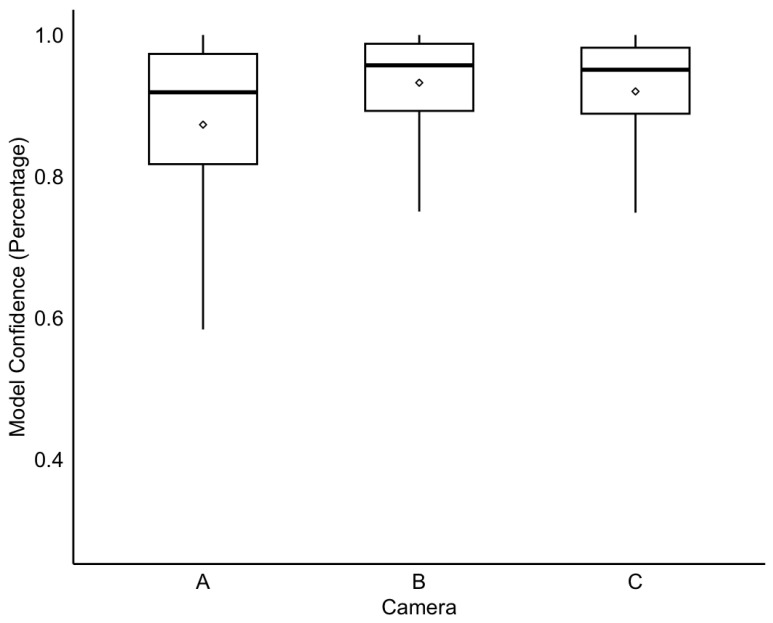
Model confidence levels across camera zones. Classification confidence scores are grouped by camera zone (A, B, and C). In each box plot, the solid horizontal line represents the median, and the open diamond indicates the mean. Whiskers extend to the minimum and maximum values.

To determine whether specific camera zones introduce variance in behaviour classification, categorizer confidence levels were compared across different viewing zones (Figure 1). The confidence levels are automatically computed by LabGym during analysis when the categorizer classifies a behaviour type and is depicted by box plots (Figure 4). There are no significant divergences in confidence levels between Camera Zone B (Mean: 93.2%, Median: 95.7%) and Camera Zone C (Mean: 92%, Median: 95.1%). However, both the mean (87.3%) and median (91.9%) values for Camera Zone A are lower compared to the other two zones, coupled with a notably lower minimum value. This difference suggests potentially reduced model performance confidence specifically for Camera Zone A. Note that all reported values exceed a 50% confidence level, as LabGym was configured to output N/A for any observations below this threshold to ensure high reliability.

### 3.4. Validation of Behavioural Categorisation and Data Divergence

**Figure 5:**
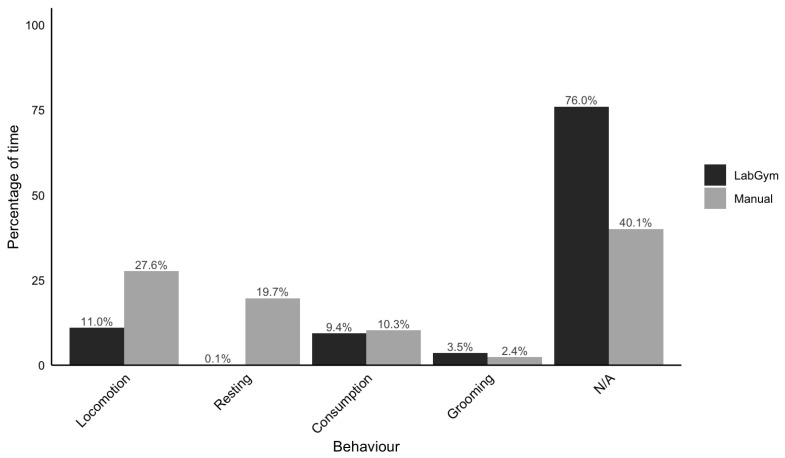
Comparison of manual versus automated behavioural classification. The percentage of total time spent on each behaviour is visualized for manual scoring based on the ethogram (grey) and classification using LabGym (black). “N/A” represents time points where the behaviour was unclassifiable or the subject was out of view.

Comparison of the manual analysis and automated categorizer analysis of red panda behaviour is depicted (Figure 5). The largest divergences are seen in the amount of unclassified behaviour (marked N/A in Figure 5), locomotion and resting. For the categorizer, 76% of the duration is unclassified compared to 40.1% in the manual analysis, which indicates a failure in the model to properly detect and classify a large portion of behaviour. Comparisons show that these discrepancies were most pronounced in locomotion and resting, where the categorizer significantly underestimated activity duration compared to manual analysis (Locomotion: 11.0% vs. 27.6%; Resting: 0.1% vs. 19.7%). The categorizer showed stronger concordance with the manual analysis for consumption (9.4% vs. 10.3%) and grooming (3.5% vs. 2.4%).

### 3.5. Analysis of Zone-Specific Activity Frequency

**Figure 6:**
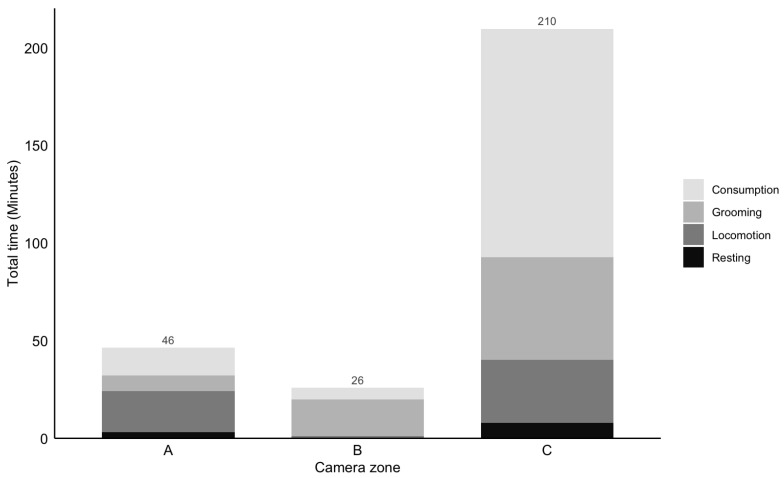
Stacked bar chart of behavioural activity duration across camera zones, derived from the automated categorization. The cumulative time (in minutes) spent on specific behaviours (Consumption, Grooming, Locomotion, Resting) is shown for Camera Zones A, B, and C. Bar segments represent the duration of each behaviour, distinguished by greyscale shading. The total duration of activity for each zone is annotated above the respective bar.

To examine space use and determine which part of the enclosure the subject most frequently occupied, a stacked bar chart of activity duration was created based on automated categorization (Figure 6).

Across the observation period, the subject spent the majority of time in Camera Zone C, with a total activity duration of 210 min. In comparison, the time spent in Camera Zones A and B was 46 min and 26 min, respectively. Furthermore, the stacked segments illustrate the distribution of specific behaviours (Locomotion, Resting, Consumption, and Grooming) within each zone.

Behavioural distribution varied notably across the three zones. Consumption was the dominant activity in Zone C, accounting for over half of the total time spent there, whereas it was nearly absent in Zone B. In contrast, Grooming was the most prominent behaviour in Zone B. Locomotion and Grooming were the most prominent activities in Zone A, while resting remained the least frequent behaviour across all zones during the examined time periods.

## 4. Discussion

The primary goal of this study was to evaluate the feasibility of using a ML approach for the continuous, automated monitoring of a red panda’s (*Ailurus fulgens*) behaviours in zoological facilities. The findings of this study provide an evaluation of the developed categorizer’s effectiveness, highlighting its strengths and limitations in classifying distinct behaviours, as well as the broader implications for applying ML in behaviour observations.

### 4.1. Technical Performance and Model Concordance

The framework of the monitoring system creates a hierarchical dependency where the categorizer’s output is strictly governed by the object detector’s recall. Consequently, any missed detections (False Negatives) result in a loss of behavioural data. This creates data loss that cannot be recovered by the categorizer, even if the latter possesses a high theoretical accuracy. The 36-percentage-point discrepancy between the categorizer’s “N/A” (76.0%) and the manual “N/A” (40.1%) possibly indicates upstream detection failures. While the categorizer has developed a robust representation of red panda posture, evidenced by high classification confidence (median > 90%), the model performance is effectively bottlenecked. In these instances, the system produces false negatives not because it misinterprets behaviour, but because environmental variables caused the detector to fail to acknowledge the subject’s presence entirely. Furthermore, the physical characteristics of the enclosure, such as large viewing distances and ground-level vegetation, may exacerbate this detection gap. The discrepancy between manual and model categorisation is most likely not a reflection of the categorizer’s inability to interpret behaviour, but rather the detector’s inability to distinguish the red panda from its complex surroundings.

Based on observations, the model struggles most in areas where the subject is partially hidden by enrichment structures or natural foliage. These findings align with Gammelgård et al. (2024), who noted that complex zoo backgrounds and obstructions by enrichment structures (e.g., ropes, nets) often complicate the automated tracking of orangutans, although their model achieved high accuracy in identifying individuals [22]. Similarly, multiple studies highlighted that fine motor movements and specific limb positioning often compromise detections in complex environments [20,29].

To address these limitations, a detection method such as a background subtraction methods could improve performance of the trained detector. Unlike feature-based detection, which relies on recognizing specific visual traits that are easily obscured by camouflage, background subtraction isolates the subject based on pixel deviations from a static reference frame. This method focuses on motion rather than morphology, potentially allowing for more consistent tracking of the red panda as it moves through dense foliage, thereby reducing False Negatives caused by the animal blending into its surroundings [30]. The potential of background subtraction methods is supported by their successful implementation in similar studies, where motion-based segmentation has proven robust in tracking subjects against complex, low-contrast backgrounds [22,31]. Background subtraction effectively detects movement but is less reliable for stationary subjects, which may be assimilated into the static background. This can be resolved by integrating complementary detection methods to maintain tracking during resting periods [32].

The categorizer was further challenged by fine-motor feeding behaviours. While the model exhibited high precision (87%) for consumption, it missed a significant number of feeding events, resulting in a low recall (56%) and the lowest overall $F_{1}$-score (68%) among all categories. Improving the recognition of these fine-motor behaviours may require higher picture resolutions, frame-rate capture or closer camera angles, an approach supported by the improved rates observed in studies utilizing facial or limb-focused datasets [20,29].

Ultimately, these results demonstrate that, while ML holds significant promise for automating behaviour monitoring, its reliability in naturalistic settings is currently limited by the environmental complexity masking the subject’s presence, rather than a lack of discriminative power in the behavioural classification itself.

### 4.2. Environmental Complexity and Spatial Analysis

While analysis of zone-specific activity frequency showed an apparent preference for Camera Zone C by the subject, this result could potentially be skewed by external factors such as varying physical features across the different camera zones. Visually, Zone C offers the most favourable conditions for automated tracking, as it contains the least amount of trees, foliage and structural obstructions. This lack of ground-level complexity reduces background noise and potentially minimizes the masking of the subject. Consequently, the apparent preference for this zone may be partially amplified by the detector’s higher success rate in simple environments. In contrast, Camera Zone B and Zone A have high densities of trees, bushes, and large logs. In these areas, the subject also frequently utilized spaces far from the lens or remains obscured by cover, leading to interruptions in tracking.

The increased duration of behaviours recorded in Camera Zone C was mainly reflected by consumption, which can potentially be due to the bamboo feeding spot being in close proximity to the camera. In addition, the recorded duration of consumption in Camera Zone A is notably lower than in Zone C, despite the presence of a primary feeding station. The feeding station in Zone A is a partially covered structure, and often obscured the subject during feeding. This environmental obstruction possibly contributes to the low recall (56%) observed for consumption, as the model struggles to acknowledge the subject’s presence when partially hidden by enrichment or natural foliage. To mitigate the difficulty of detection in key areas of interest, installing supplementary cameras in closer proximity to critical areas, such as feeding stations and common sleeping sites, would be beneficial to divert physical obstructions and improve detection consistency.

However, the recorded duration may also indicate a biological preference for this area. Zone C provides both feeding spots and access points for arboreal movement, represented by greater durations in all behaviours. Furthermore, Camera Zone C is the area least frequented by the cranes present in the enclosure, while also being the furthest point from the open-window viewing platform for guests, suggesting that the subject may seek out Zone C to avoid disturbance.

### 4.3. Prospects of Automated Monitoring

This study highlights the significant potential of automated systems for facilitating long-term, continuous behavioural monitoring, in alignment with previous research [18,20,21]. The primary advantage of automated tracking is its capacity to capture the red panda’s full 24 h cycle, accounting for activity peaks at dawn, dusk, and midnight—periods which are typically missed by traditional daytime keeper observations [15]. Continuous monitoring is required to establish a baseline of the behaviour types and activity/inactivity of the animal. This allows both researchers and animal care staff to differentiate between natural variations in behaviours, influenced by environmental factors such as temperature [15], or behaviour changes triggered by external stressors [14,24]. Consequently, any future implementation of ML for automated behaviour analysis must prioritize the robust detection and classification of both active and inactive states to accurately detect abnormal changes in behaviour.

A primary limitation of application of automated monitoring is the computational demand and time requirement to process video material using the model. At present, the analysis of extensive footage remains computationally intensive, requiring high-performance hardware that can be prohibitively expensive or resulting in extended processing times on standard systems. This poses a barrier to real-time application. Furthermore, the study’s reliance on specific subjects for model training could limit the application of the model on individuals with other phenotypic traits and its ability to differentiate between individuals. Gammelgård et al. (2024) [22] noted that models trained on specific individuals often see a decrease in accuracy when applied to new subjects. Future studies should aim to train models on multi-individual datasets across varying seasons to account for changes in foliage, lighting conditions and pelage.

Future research must prioritize reducing the N/A rate through a combination of hardware, algorithmic, and software optimizations. Future implementations should prioritize placing cameras in closer proximity to key areas of interest, such as feeding stations and common resting places. This setup could be enhanced by integrating thermal imaging, which would address the camouflage issue entirely and improve detection during the red panda’s active nocturnal/crepuscular periods. Algorithmic improvement efforts should focus on improving the object detector’s robustness against complex backgrounds, and improving differentiation of fine motor movements.

Similar studies indicate that the analytical capabilities of the system could be improved by supplementing or transitioning to software frameworks specifically designed for pose estimation and tracking, such as the software SLEAP (Social LEAP Estimates Animal Poses) [22,33,34]. While LabGym relies on the detection of the subject’s entire silhouette, pose estimation focuses on the localization of specific anatomical landmarks (e.g., the base of the tail, limb joints, and head) [33]. In complex environments where full-body detection is frequently compromised, pose estimation represents a potential alternative, allowing for behavioural analysis based on visible anatomical landmarks rather than requiring an unobstructed view of the entire subject.

## 5. Conclusions

This study demonstrates that further development of the categorizer can enable automated ML monitoring, providing the continuous data on captive red panda behaviour necessary to inform evidence-based welfare management strategies. The analysis indicates that the behaviour categorizer is effective, showing robust performance and high confidence when the subject is successfully located. However, the system is limited by the object detector, which struggled to consistently identify the red panda due to foliage obstruction and its natural camouflage with surroundings. At its current stage, these limitations may restrict the model’s ability to supplement the zookeepers’ manual monitoring of red panda behaviour. However if these limitations are addressed, ML shows high potential as an automatic monitoring tool of red panda behaviour. Future implementations should prioritize robust detection and categorisation, potentially by incorporating thermal imaging, background subtraction methods to filter environmental noise, or pose-estimation frameworks, to ensure the continuous data necessary for reliable behaviour assessments.

## Figures and Tables

**Table 1:** Ethogram of target behaviours. Descriptions and definitions of the specific behaviours observed in the red panda subject, used for the classification of training data and manual evaluation. Based on ethogram by Bugler et al. 2023 [15].

Behaviour	Definition
**Locomotion**	Quadrupedal walking, running, or trotting on the ground, or horizontal logs/branches that are at ground level. Climbing, descending, leaping, or moving along elevated platforms, trees, or horizontal branches above ground level.
**Resting**	Lying down, sitting, or adopting a curled posture on the ground or an elevated platform. Characterized by minimal to no body movement.
**Consumption**	Consumption of bamboo leaves or feeding pellets. Includes holding food with paws and chewing.
**Grooming**	Cleaning coat or body by licking or using paws to rub the head/face. Scratching body by using paws.
**N/A**	Time points at which subject behaviour was unclassifiable or the subject was out of view.

**Table 2:** Inter-rater reliability results expressed as Intraclass Correlation Coefficients (ICCs) for each behavioural category.

Behaviour	ICC
Locomotion	0.927
Resting	0.905
Consumption	0.846
Grooming	0.965

**Table 3:** Performance of the red panda behaviour categorisation model. The table shows precision, recall, F_1_-scores, and support for each behaviour. Overall accuracy of the model across all classes was 76%.

Behaviour	Precision	Recall	F_1_-Scores	Support
**Locomotion**	0.69	0.94	0.8	36
**Resting**	0.84	0.84	0.84	31
**Consumption**	0.87	0.56	0.68	59
**Grooming**	0.7	0.85	0.77	39

**Figure A1:**
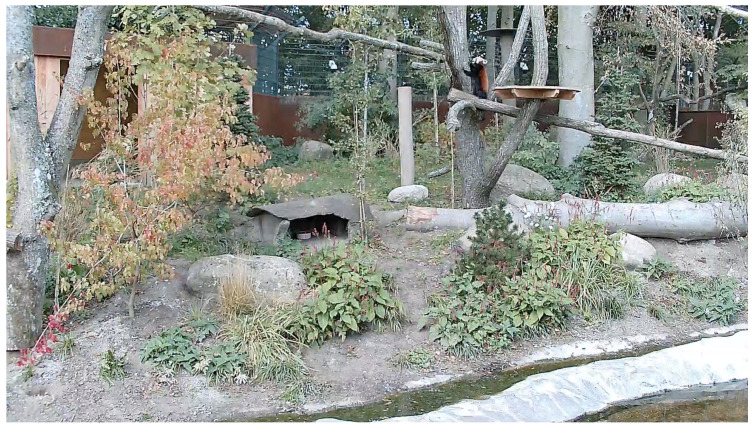
Environmental complexity within the field of view of camera A. The snapshot illustrates the habitat layout, including climbing structures, water features (bottom right), vegetation, and the ground-level feeding station. The red panda is visible on the climbing structure (right). This camera angle provided the raw footage for the zonal classification in zone A (Figure 1).

**Figure A2:**
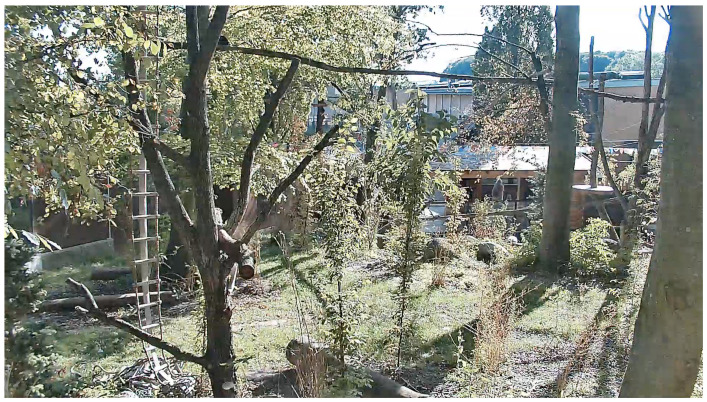
Environmental complexity within the field of view of camera B. The snapshot illustrates the habitat layout, characterized by dense vegetation, arboreal pathways, and a vertical rope ladder (left). The red panda is visible on the climbing structure in the upper left corner. This camera angle provided the raw footage for the zonal classification in zone B (Figure 1).

**Figure A3:**
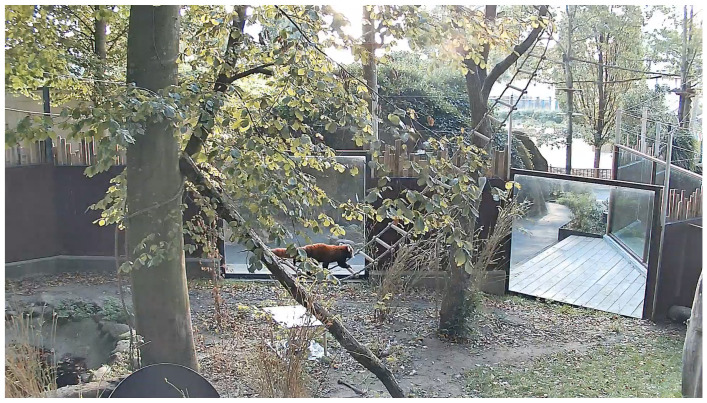
Environmental complexity within the field of view of camera C. The snapshot illustrates the habitat layout, distinguished by the glass visitor viewing area, water features, and horizontal arboreal pathways. The red panda is visible crossing a rope ladder in the center of the frame. This camera angle provided the raw footage for the zonal classification in zone C (Figure 1).

## Data Availability

The data presented in this study are available on request from the corresponding author.

## Appendix A

To ensure comprehensive monitoring of the subject’s activity, three cameras were positioned to capture distinct zones (A, B, and C) within the red panda enclosure. These vantage points were selected to maximize visibility of key behavioural enrichment areas, including climbing structures and feeding stations. The field of view of three camera units overlooked the enclosure (Figure 1). The raw video feeds served as the input for the classification model.

## Appendix B

To establish a robust detection model capable of distinguishing the subject from the complex background, raw video frames were manually annotated using Roboflow. As illustrated in Figure A4, polygon annotation was employed rather than bounding boxes. This method involves tracing the precise contours of the subject (Red Panda), effectively isolating the animal from environmental noise such as foliage, shadows, and enclosure structures. The annotated images served as the training input for the detector module within LabGym.

To train the LabGym categorizer model, a ground truth dataset was established through manual sorting. Video segments were reviewed and classified according to the pre-defined ethogram (Table 1). Figure A5 illustrates representative behaviour examples: locomotion, resting, and grooming. The left panels display the video segment used for manual identification, while the right panels depict the corresponding contour generation performed by the software LabGym during the training phase. These visual signatures allow the model to learn the specific spatiotemporal features associated with each behaviour.
